# Tics severity in Tourette syndrome associated with higher glutamatergic activity in the anterior cingulate cortex

**DOI:** 10.1093/braincomms/fcag212

**Published:** 2026-06-17

**Authors:** Mathilde Boussac, Anne-Sophie Salabert, Estelle Harroch, Claire Thalamas, Christophe Arbus, Yves Chaix, Anne-Laure Toureille Pouget, Pierre Payoux, Christine Brefel-Courbon

**Affiliations:** Toulouse Neuroimaging Centre (ToNIC), Inserm UMR1214, University of Toulouse III (UPS), 31059 Toulouse, France; Toulouse Neuroimaging Centre (ToNIC), Inserm UMR1214, University of Toulouse III (UPS), 31059 Toulouse, France; Department of Radiopharmacy, Toulouse University Hospital, 31059 Toulouse, France; Department of Clinical Pharmacology and Neurosciences, Parkinson’s Expert Centre, Toulouse University Hospital, NeuroToul COEN (Centre of Excellence in Neurodegeneration), 31059 Toulouse, France; NS-PARK/FCRIN Network, France; Clinical Investigation Centre (CIC 1436), Department of Clinical Pharmacology, Toulouse University Hospital, 31059 Toulouse, France; Clinical Investigation Centre (CIC 1436), Department of Clinical Pharmacology, Toulouse University Hospital, 31059 Toulouse, France; Toulouse Neuroimaging Centre (ToNIC), Inserm UMR1214, University of Toulouse III (UPS), 31059 Toulouse, France; Department of Psychiatry, Toulouse University Hospital, Purpan University Hospital Centre, 31059 Toulouse, France; Toulouse Neuroimaging Centre (ToNIC), Inserm UMR1214, University of Toulouse III (UPS), 31059 Toulouse, France; Department of Paediatric Neurology, Children's Hospital, Toulouse University Hospital, 31059 Toulouse, France; Department of Paediatric Neurology, Children's Hospital, Toulouse University Hospital, 31059 Toulouse, France; Midi-Pyrénées Autism Resource Centre, 31300 Toulouse, France; Toulouse Neuroimaging Centre (ToNIC), Inserm UMR1214, University of Toulouse III (UPS), 31059 Toulouse, France; Department of Nuclear Medicine, Toulouse University Hospital, Toulouse Cedex 31059, France; Toulouse Neuroimaging Centre (ToNIC), Inserm UMR1214, University of Toulouse III (UPS), 31059 Toulouse, France; Department of Clinical Pharmacology and Neurosciences, Parkinson’s Expert Centre, Toulouse University Hospital, NeuroToul COEN (Centre of Excellence in Neurodegeneration), 31059 Toulouse, France; NS-PARK/FCRIN Network, France

**Keywords:** Tourette syndrome, PET, glutamate, NMDA receptors, anterior cingulate cortex

## Abstract

Tourette syndrome is a neuropsychiatric disorder whose physiopathology is still poorly understood. It involves alterations in the dopaminergic system within the cortico-basal ganglia-thalamo-cortical loops although several hypotheses from the literature implicate the glutamatergic system. Our objective was to study the activity of NMDA receptors (NMDARs) in vivo in patients with Tourette syndrome compared to healthy controls. PET imaging was used in patients with Tourette syndrome (*n* = 12) and healthy controls (*n* = 12) with a new radioligand, the [^18^F]-FNM, which binds to activated NMDARs. Clinical and behavioural assessments were also performed on patients to evaluate tics severity (YGTSS), obsessive–compulsive disorder (Y-BOCS), impulsivity (BIS-11) and anxio-depressive state (HAD). PET activity was compared between groups in different ROIs. Correlations were made between PET activity and clinical/behavioural scores in patients with Tourette syndrome. Significant differences in NMDAR activity were found between patients with Tourette syndrome and controls in the right and left ACC, the right caudate nucleus, the right olfactory cortex and the left paracentral lobule: patients with Tourette syndrome exhibited higher NMDAR activity in these ROIs. Moreover, this hyper-NMDAR activity in the ACC correlated positively and significantly with YGTSS scores in patients with Tourette syndrome. Patients with Tourette syndrome showed higher NMDAR activity in the ACC, caudate nucleus, paracentral lobule and olfactory cortex. This presumed hyper-glutamatergic activity may indicate a hyper-activation of the direct striatal pathway leading to the onset of tics, in addition to the supposed hypo-activation of the indirect striatal pathway related to hypo-GABAergic state reported in the literature. Tourette syndrome would therefore be associated with an imbalance between excitatory and inhibitory influences within the cortico-basal ganglia-thalamo-cortical circuit.

## Teaser

This study aimed to evaluate the activity of glutamatergic receptors (NMDAR) in Tourette syndrome using PET imaging. Boussac *et al*. reported that patients with Tourette syndrome presented a higher NMDAR activity compared to matched healthy controls, in several cortical regions. Specifically, the hyper-NMDAR activity in the anterior cingulate cortex was correlated with tics severity in Tourette syndrome, probably reflecting a hyper-activation of the direct striatal pathway leading to tics emergence.

## Graphical Abstract

**Graphical Abstract:**
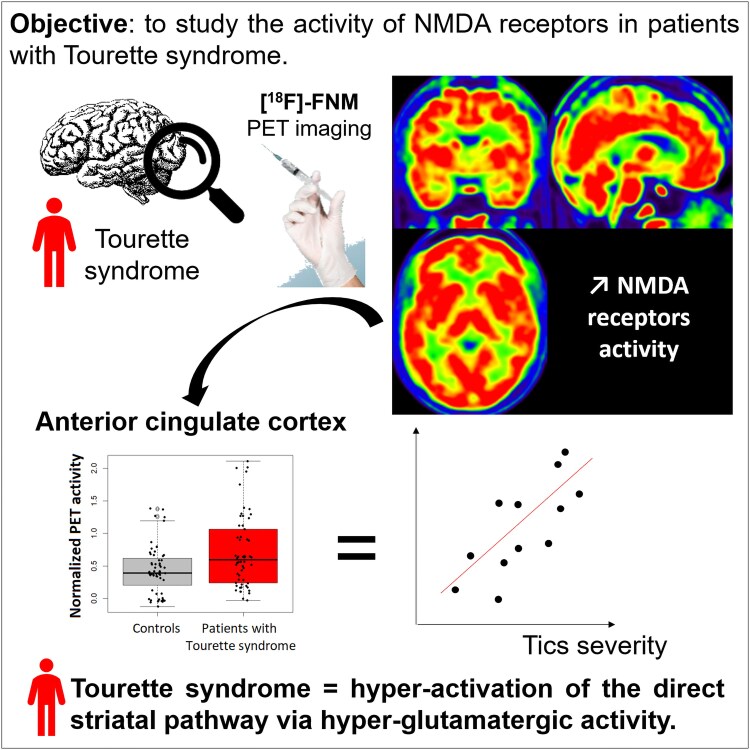

## Introduction

Gilles de la Tourette syndrome (TS) is a neuropsychiatric disorder characterized by motor and phonic tics emerging during childhood.^1-3^ Its prevalence is about 0.7% in children and around a global incidence of 0.5% in adolescents.^4-6^ Tics differ in forms and severity and can have a significant impact on quality of life and socio-professional life. Moreover, TS is often associated with psychiatric comorbidities such as obsessional-compulsive disorders (OCDs), hyperactivity [attention-deficit and hyperactivity disorders (ADHDs)], autism and anxio-depressive state.^3^

As tics are repetitive involuntary movements, they are supposedly related to dysfunction in the cortico-basal ganglia-thalamo-cortical (CBGTC) loops implicated in movement disorders.^7-9^ In this complex circuitry, many neurotransmitters could be involved in the pathophysiology of tics such as dopamine, GABA, glutamate etc..^8^ Thus far, the dopaminergic system has been the most widely studied in TS, and some pharmacological and genetic evidence exists, as well as animal models, implying a hyper-dopaminergic activity in patients with TS.^3,8,10-13^ Indeed, neuroleptics, such as the dopaminergic antagonist aripiprazole, are usually effective at improving patient tics.^3^

Moreover, an anti-epileptic agent acting as a glutamatergic antagonist of AMPA receptors, topiramate, is also useful in the treatment of TS, suggesting an implication of the glutamatergic system in TS.^14,15^ Its involvement in the CBGTC loops means the glutamatergic system interacts with the dopaminergic pathway.^16^ Indeed, glutamate is a ubiquitous neurotransmitter and one of the most widely used neurotransmitters in the central nervous system. It mainly serves as a modulator of other neurotransmitters through interneurons.^17-19^ Hence, there are some genetic, post-mortem, imaging and pharmacologic studies that support evidence of a glutamate implication in TS.^8^ For example, studies have demonstrated that tics severity was reduced in a transgenic model of rodents inducing glutamatergic hyperactivity after administration of drugs diminishing cortico-striatal glutamatergic transmission.^20-22^ Post-mortem studies also revealed decreased glutamate levels in the globus pallidus and the pars reticula of the substantia nigra of patients with TS.^23^ Genetic studies have also identified a missense variant of the glial glutamate 1 transporter (*SLC1A3*) in some patients with TS.^24-26^

Still on the topic of CBGTC loops, the literature has also highlighted a failure in the top-down cortical control within motor loops which could explain the emergence of tics in TS.^27,28^ Indeed, a dysfunction in cortical inhibition mainly at the prefrontal and premotor regions was shown in an MRI study.^29^ Thus, the GABAergic system could also be involved in TS. An animal model of TS has been established using local microinjections of a GABA antagonist into the striatum.^30^ In patients with TS, post-mortem studies have revealed a loss of GABAergic interneurons in the striatum^31^ and a reduction in GABA concentrations in the primary sensorimotor cortex correlated with the severity of motor tics.^32^ In addition, imaging studies have shown alterations in GABA binding in several regions (striatum, globus pallidus, thalamus, posterior cingulate cortex etc.) in patients with TS.^33^

Additionally, since dysfunctions within the basal ganglia circuit are not sufficient to explain some characteristics of TS (predominance of motor tics within some body parts, presence of vocal tics, natural history of tics etc.^34,35^), other cerebral systems may also be involved such as ‘social decision-making network’^34,35^ and are currently under discussion in the literature.

To directly study brain dysfunctions, different imaging approaches are available such as PET, which allows us to functionally investigate different neurotransmitter systems in vivo in the brain. To explore part of the glutamatergic system, the [^18^F]-FNM (fluoroethylnormemantine) radiotracer was recently developed by our group.^36-38^ This radioligand is derived from memantine, a glutamatergic antagonist, which binds to the phencyclidine (PCP) intra-canal site of NMDARs.^38^ This PCP site is known to be mainly accessible when NMDARs are depolarized and activated.^39,40^ Consequently, the [^18^F]-FNM is believed to bind to activated NMDARs, allowing visualization of the NMDAR activity in vivo. Indeed, the last study on pharmacological characterization of the [^18^F]-FNM in rats confirmed the ability of this tracer to evaluate activation of NMDARs with an affinity of Ki = 3.39e10−5 and a good selectivity due to its binding on opened NMDARs.^38^ Therefore, the [^18^F]-FNM can be considered as an uncompetitive antagonist of NMDARs, tracking brain regions with important activation of NMDARs. In fact, the [^18^F]-FNM affinity to NMDAR was between ∼7 and ∼10-fold higher compared to other binding sites such as opioid receptors (IC_50_ NMDAR PCP site in forebrain = 1.3e10^−5^ versus IC_50_ opioid receptor = 9.6e10^−5^ and IC_50_ µ opioid receptor = 1.4e10^−4^).^38^

Using this [^18^F]-FNM radioligand, our pilot study (GlutaTour) (clinicaltrials.gov: NCT03681795) aimed to visualize and compare NMDAR activity in vivo in patients with TS and healthy controls using PET. Secondary objectives were to investigate the correlations between NMDAR activity and clinical and behavioural symptoms in patients with TS.

## Materials and methods

### Patient consent

All subjects gave their informed and written consent, according to the Declaration of Helsinki.

#### Participants and clinical assessment

Twelve patients diagnosed with TS according to the *Diagnostic and statistical manual of mental disorders*, 5th ed criteria were included in Toulouse University Hospital Centre (France). All patients were adults (≥18 years old) and treated with aripiprazole for at least 3 months. Patients were excluded if they had another neurological or psychiatric disease interfering with examination results, MRI contraindication, troublesome tics of the head and/or shoulders compromising imaging examinations or if they were pregnant or breastfeeding. Healthy controls were aged ±3 years-, sex- and manual laterality matched to patients with TS. The Mini-International Neuropsychiatric Interview was used on healthy controls to exclude subjects with any neuropsychiatric disorders. Anxio-depressive [assessed using the Hospital Anxiety and Depression scale (HAD) > 7] and impulsive subjects [evaluated using the Barratt Impulsivity Scale-11 items (BIS-11) ≥ 72^41^] were not included. Controls treated by neuroleptics or any treatment susceptible to interfere with NMDARs or who had a family member with TS were also excluded.

The Yale Global Tic Severity Scale (YGTSS) and Yale-Brown Obsessive Compulsive Scale (Y-BOCS) were used on patients with TS; HAD and BIS-11 scales were performed for TS and control subjects. Single MRI and PET measurements were carried out for each subject.

All subjects gave their informed and written consent, and the GlutaTour study was approved by an Ethics Committee: the Comité de Protection des Personnes (CPP: Committee for the Protection of Persons) Sud-Ouest et Outre-Mer I ou II (CPP: RC31/15/7836, EudraCT Number: 2017-000816-40). All methods were carried out in accordance with relevant guidelines and regulations.

#### Radiochemistry

[^18^F]-FNM was prepared in line with a previously described method.^36^ In brief, [^18^F]-FNM was produced by nucleophilic substitution using 1-[N-(tert-butyloxy)carbamoyl]-3-(tosyl)ethyl-adamantane (M2i; Rest Therapeutics) as the precursor on a Raytest® module and an AIO trasis® module. After complete removal of the solvent by azeotropic drying, the precursor was added to the reaction vial and heated for 20 min at 125°C. The reaction mixture was then cooled and added to the hydrolysis solution and heated for 10 min at 110°C, causing hydrolysis of the BOC (tert-butoxycarbonyle) group. The reaction mixture was then neutralized by adding 6 N NaOH and 0.5 M trisodium citrate solutions. Pre-purification was achieved using a Sep-Pak cartridge (waters C18 Plus). The lipophilic compound trapped in the cartridge was eluted with 2 mL of ethanol. High-performance liquid chromatography (HPLC) purification was carried out in a semi-preparative column (Cluzeau Info Labo Stability Basic C-18 CIL; 250 × 10 mm, particle size 5 μm), with a mobile phase consisting of ethanol absolute/sodium acetate (0.1 M) mixture (45/55; v/v). The [^18^F]-FNM retention time was 15 min, with a flow rate of 2 mL/min. The radioligand was obtained in high radiochemical purity (>95%) and had a molar activity >25 GBq/µmol at the time of injection. No chemical impurities were detected during HPLC quality control. [^18^F]-FNM is a new experimental drug and was approved by the French Health Authorities (ANSM: French National Agency for Medicines and Health Products Safety) for first human use after validation by an Investigational Medical Product Dossier. Description of this radiotracer, its pharmacological characterization and evaluation of its bio-distribution can be found in previous studies on animal models.^36-38^

#### Image acquisition

MRI data were acquired at the Toulouse Neuroimaging Centre (ToNIC) MRI technical platform with an MRI 3T Philips ACHIEVA scanner using a dStream head coil (32 channels). Structural MRI included an acquisition whole-head in three dimensions, in sagittal section and weighted in T1 with the following parameters: MPRAGE, repetition time/echo time = 7.4/3.5 ms, flip angle = 8°, field of view = 240 × 240 × 180 mm, isotopic voxel size = 1 mm^3^. During MRI acquisition, participants were placed in supine position, their arms to the sides. They were instructed to keep their eyes opened, remain still, relax and not think of anything in particular while staying awake.

Following the MRI examination, a brain PET scanner was performed using hybrid PET/CT tomograph (Siemens Biograph TruePoint 6.0). An intravenous bolus injection of [^18^F]-FNM (3.5 MBq/kg) was carried out (mean dose of 280 ± 53 MBq). After 30 min of rest after the injection, ‘low dose’ scanner acquisitions were realized in supine position, with the head held in a headrest. After completion of scanner acquisition, PET dynamic images were acquired within 30 to 55 min post-injection, according to kinetics evaluation in the rats where uptake in the rat brain was found constant after 40 min of injection.^36^ Dynamic data were reconstructed into five timeframes (5 × 5 min). All corrections (attenuation, radioactive decay, random, scatter-coincidences and a partial-volume correction) were incorporated in an iterative OSEM reconstruction (3 iterations, 21 sub-sets).

#### Image processing

PET imaging processing and quantification were performed using PMod software (version 3.9) separately for each subject. The pipeline included the following steps:

(1) realignment of the five dynamic frames to the first frame using motion correction; (2) rigid co-registration of the realigned frames to the subject’s anatomical T1-weighted MRI; (3) segmentation of the five co-registered timeframes using the automated anatomical labelling (AAL-merged) atlas (67 ROIs) in the subject native-space based on the MRI; and (4) extraction of [^18^F]-FNM-absorption values [mean time activity curves (TACs) and standard deviation] for each ROI and subject. In parallel, grey matter (GM) and white matter (WM) masks were segmented on SPM12 (statistical parametric mapping) toolbox in MatLab software (version R2023a) for each subject.^42^ These two masks were merged to extract GM + WM activity values (mean TACs and standard deviation) for each subject back in PMod. Finally, PET activity of each timeframe was standardized according to the GM + WM activity using intra-subject normalization: **normalized PET activity = (**$\frac{ROI\;mean\;activity\;-\;whole\;brain\;mean\;activity}{whole\;brain\;standard\;deviation}$**)**. GM + WM standardization was selected due to the ubiquitous distribution of NMDAR in the brain,^40,43,44^ while intra-subject normalization is important to correct for individual heterogeneity.

During the first step, data quality was assessed by visually inspecting the realignment of the five PET frames to ensure that no significant head motion had occurred during acquisition. The accuracy of the realignment, co-registration and segmentation steps was also verified for each subject. All datasets met quality criteria and were retained for subsequent analyses.

### Statistical analyses

Demographical, clinical and behavioural data were expressed as mean and standard deviation for the quantitative variables; and headcount and percentage for the categorical ones. Based on variable characteristics and distribution, differences between groups were assessed using two-sample Student’s t-tests or Mann-Whitney U tests. Categorical variables were analysed using Chi^2^ tests.

For each subject, normalized PET activity of the five timeframes was used for all subsequent analyses.

First, for the main objective, normalized PET activity of each ROI taken together was compared between groups (patients with TS versus controls) [ANOVA with groups as explicative factor, adjusted by treatment dose (aripiprazole)]. To go a step further and study which ROI had specifically a different PET activity in patients with TS, ROI-wise comparisons of [^18^F]-FNM PET activity were performed among groups [ANOVAs with groups as explicative factor, adjusted by treatment dose (aripiprazole)]. Mean differences and 95% CI were also calculated. To correct for multiple comparisons, false discovery rate (FDR) *P*-values were calculated.

Afterwards, associations between normalized [^18^F]-FNM PET activity in specific ROIs and clinical/behavioural measures (including age, treatment dose, total and sub-scores of YGTSS, Y-BOCS, HAD and BIS-11) were examined in patients with TS using Spearman correlation analyses. Correlations analyses were restricted to ROIs showing significant between-group differences in PET activity. FDR correction was applied to control for multiple comparisons and to ensure the pertinence of significant effects.

Statistical analyses were performed on RStudio software (version 2022.12.0). Significance was set at *P* < 0.05. For correlations, only significant FDR-corrected *P*-values and coefficients ≥ |0.6| were considered relevant.

## Results

### Participant characteristics

Population characteristics are presented in Table 1. Patients with TS were 28.7 ± 10.1 years old, with a mean severity of tics of 49.8 ± 13.8 (YGTSS total score) and were all treated by aripiprazole (mean dose of 7.5 ± 3.0 mg/day). Other drugs included trihexyphenidyl (*n* = 1), melatonin (*n* = 1), sertraline (*n* = 1) and venlafaxine (*n* = 1). Three patients with TS (25%) had an OCD as comorbidity. Healthy controls were 31.3 ± 10.4 years old.

**Table 1:** Description of the population

Variable	Patients with TS (*n* = 12)				Healthy controls (*n* = 12)				
	Mean	SD	Min	Max	Mean	SD	Min	Max	*P*-value^a^
Sex (men; women)	10; 2	/	/	/	10; 2	/	/	/	1^$^
Age (years)	28.7	10.1	18	51	31.3	10.4	20	52	0.35
Weight (kg)	84.0	14.9	61	110	76.6	15.9	60	110	0.25
Height (cm)	175.3	7.0	165	188	174.1	5.8	165	184	0.64
Manual laterality (right; left)	10; 2	/	/	/	10; 2	/	/	/	1^$^
Treatment dose [aripiprazole (mg/day)]	7.5	3.0	2.5	12.5	/	/	/	/	/
YGTSS Total/100	49.8	13.8	21	74	–	–	–	–	–
YGTSS Motor/25	13.0	2.9	8	18	–	–	–	–	–
YGTSS Vocal/25	9.3	4.9	0	19	–	–	–	–	–
YGTSS Motor + Vocal/50	22.3	6.3	11	34	–	–	–	–	–
YGTSS Impairment/50	27.5	8.7	10	40	–	–	–	–	–
Y-BOCS Total/40	6.2	6.9	0	18	–	–	–	–	–
Y-BOCS Obsessions/20	2.6	3.9	0	12	–	–	–	–	–
Y-BOCS Compulsions/20	3.5	4.3	0	12	–	–	–	–	–
HAD Total/42	12.4	7.2	3	24	2.8	2.2	0	6	0.0007*
HAD Anxiety/21	8.3	4.2	3	16	2.3	2.0	0	6	0.0009*
HAD Depression/21	3.8	3.6	0	12	0.5	0.9	0	3	0.002*
BIS-11 Total/120	67.5	12.9	44	91	55.3	8.5	40	70	0.01*
BIS-11 Planning difficulties/44	24.8	4.6	16	34	21.6	3.5	16	29	0.07
BIS-11 Motor impulsivity/44	23.5	5.6	16	33	18.8	3.9	13	25	0.03*
BIS-11 Cognitive impulsivity/32	19.2	4.0	12	25	14.9	2.6	10	17	0.007*
Injected dose (MBq)	301.1	48.8	228	384	258.6	50.5	199	344.8	0.05
Injected dose-to-weight ratio (MBq/kg)	3.61	0.37	3.04	4.44	3.39	0.29	2.77	3.75	0.13

TS, Tourette syndrome; YGTSS, Yale Global Tic Severity Scale; Y-BOCS, Yale-Brown Obsessive Compulsive Scale; HAD, Hospital Anxiety and Depression Scale; BIS-11, Barratt Impulsivity Scale-11 items; ^a^Two-sample tests comparing patients with TS and healthy controls, ^$^except for the quantitative variables where Chi^2^ tests were done: significant *P*-values in bold*.

There were no demographic differences between groups in terms of sex, age, weight, height and manual laterality. Patients with TS demonstrated significantly higher scores in the HAD and BIS-11 scales (total scores, HAD anxiety, HAD depression, BIS-11 motor impulsivity and BIS-11 cognitive impulsivity sub-scores).

### [^18^F]-FNM PET findings in TS

**Figure 1:**
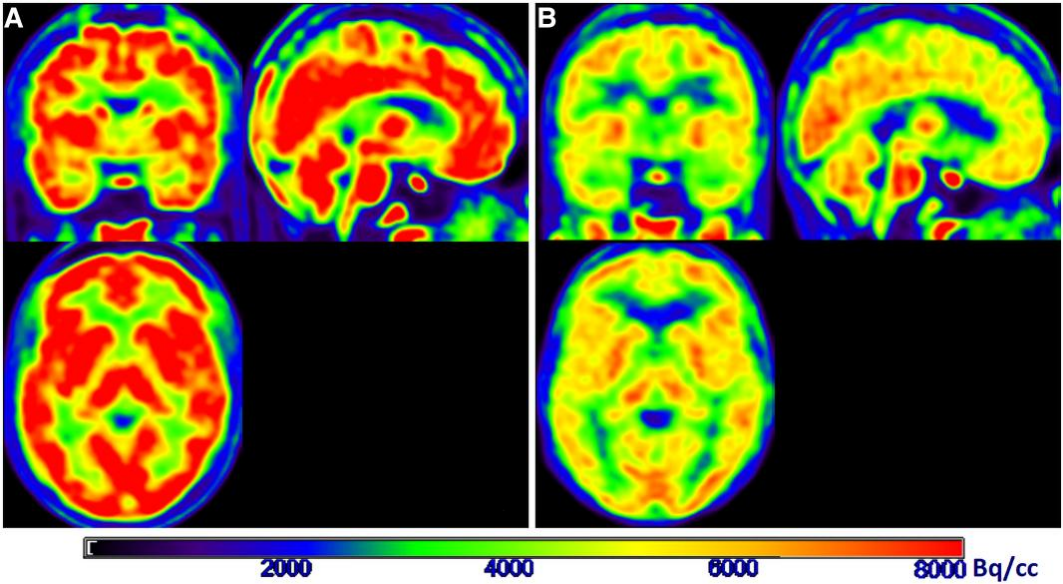
**Comparisons of PET activity between two paired subjects.** PET cerebral imaging of **(A)** a patient with TS and **(B)** its paired healthy control in a normalized template. Scale from blue to red represents the intensity of PET uptake, which is the intensity of NMDAR activity in PET imaging for both subjects in Bq/cc. The injected weight-to-dose ratio was of 3.4 MBq/kg for the patient with TS and 3.6 MBq/kg for its paired healthy control. Frames A and B: x, y, z = 79, 113, 71. ANOVA of the difference between groups (*n* = 12 patients with TS versus *n* = 12 healthy controls): *P* = 1.2 × 10^−5^, F = 19.12, df between groups = 1 and df within groups = 22.

When comparing both groups, there was a considerable effect of the group in terms of normalized PET activity for all ROIs taken together (ANOVA: *P* = 1.2 × 10^−5^ and *F* = 19.12): patients with TS had an overall higher PET activity compared to healthy controls (Fig. 1 and Supplementary Fig. 1).

**Figure 2:**
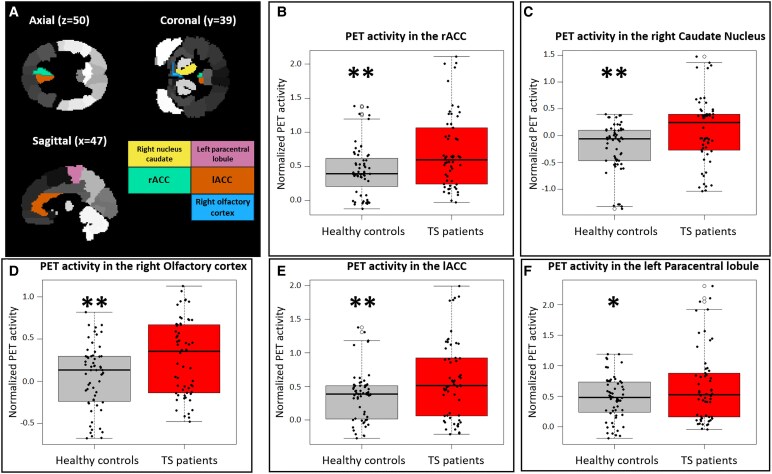
**Comparisons of PET activity between groups according to ROIs. (A)** Representation of the ROIs with hyper-NMDAR activity in an AAL atlas: coloured overlays correspond to the right caudate nucleus in yellow, the left paracentral lobule in pink, the rACC in green, the lACC in orange and the right olfactory cortex in blue. Boxplots with individual values of the normalized PET activity from the five timeframes (that is five datapoints for each subject corresponding to the five values of the normalized PET activity) of rACC (**(B)**—*P*-value = 0.002 and F-value = 10.54); the right caudate nucleus [**(C)**—*P*-value = 0.002 and F-value = 9.99[; the right olfactory cortex [**(D)**—*P*-value = 0.003 and F-value = 9.12[; lACC [**(E)**—*P*-value = 0.007 and F-value = 7.50] and the left paracentral lobule [**(F)**—*P*-value = 0.04 and F-value = 4.32]. Grey boxplots correspond to the healthy controls and red boxplots to the patients with TS. ANOVAs with groups as explicative factor, adjusted by treatment dose (*n* = 12 patients with TS versus *n* = 12 healthy controls): **P-value < 0.01, *P-value < 0.05, df between groups = 1 and df within groups = 22.

Compared to healthy controls, patients with TS exhibited significantly higher PET activity in the right anterior cingulate cortex (rACC), the right caudate nucleus, the right olfactory cortex, the left anterior cingulate cortex (lACC) and the left paracentral lobule (ANOVAs adjusted with treatment dose: *P*-values < 0.05). Using FDR correction, three of these ROIs still tended to be significant: the rACC, the right caudate nucleus and the right olfactory cortex (FDR-corrected *P*-values = 0.07). Comparisons of ROIs activity between groups are presented in Table 2 along with FDR *P*-values, mean differences and 95% CI, and significant results are presented in Fig. 2.

**Table 2:** Comparisons of ROI-normalized PET activity between groups

ROIs	Hemisphere	MNI coordinates (*x y z*)	Mean normalized PET activity of the five timeframes (intrasubject normalized TAC values)		Difference between groups		ANOVAs between groups adjusted with treatment dose	
			Patients with TS (*n* = 12)	Healthy controls (*n* = 12)	Mean differences	95% CI	*P*-value of the group effect	FDR-corrected *P*-value of the group effect
Anterior cingulate cortex (rACC)	R	(−9 28 7)	0.71	0.44	−0.27	[−0.44; −0.1]	0.002 *	**0**.**07**
Caudate nucleus	R	(−13 1 4)	0.1	−0.21	−0.31	[−0.51; −0.11]	0.002 *	**0**.**07**
Olfactory cortex	R	(−16 18 −13)	0.28	0.05	−0.23	[−0.38; −0.07]	0.003 *	**0**.**07**
Anterior cingulate cortex (lACC)	L	(7 25 8)	0.59	0.35	−0.24	[−0.42; −0.06]	0.007 *	0.12
Paracentral lobule	L	(9 −27 64)	0.66	0.47	−0.19	[−0.37; 0]	0.04 *	0.54
Postcentral gyrus	L	(39 −24 46)	0.73	0.56	−0.17	[−0.35; 0.01]	0.06	0.66
Supplementary motor area	R	(−9 −2 60)	0.78	0.63	−0.15	[−0.31; 0.01]	0.07	0.66
Postcentral gyrus	R	(−44 −63 37)	0.73	0.58	−0.15	[−0.32; 0.03]	0.10	0.72
Angular gyrus	R	(−40 −27 46)	0.9	0.74	−0.16	[−0.36; 0.03]	0.09	0.72
Olfactory cortex	L	(13 17 −13)	0.33	0.2	−0.13	[−0.3; 0.03]	0.11	0.72
Posterior cingulate cortex	R	(−9 −47 16)	0.05	0.32	0.27	[−0.08; 0.6]	0.12	0.75
Supramarginal gyrus	L	(54 −37 27)	0.77	0.62	−0.15	[−0.37; 0.06]	0.14	0.78
Lingual gyrus	R	(−23 −66 −7)	0.91	0.77	−0.14	[−0.34; 0.06]	0.15	0.78
Parietal lobe	R	(−34 −56 56)	0.67	0.56	−0.11	[−0.28; 0.05]	0.16	0.78
Caudate nucleus	L	(11 0 4)	0.15	−0.02	−0.17	[−0.4; 0.07]	0.17	0.79
Parietal lobe	L	(33 −52 56)	0.82	0.7	−0.12	[−0.3; 0.07]	0.20	0.82
Middle frontal	L	(26 28 19)	0.7	0.58	−0.12	[−0.32; 0.08]	0.23	0.82
Posterior cingulate cortex	L	(7 −43 17)	0.29	0.5	0.21	[−0.15; 0.57]	0.23	0.82
Putamen	L	(21 −1 0)	1.37	1.23	−0.14	[−0.4; 0.12]	0.28	0.82
Precentral gyrus	L	(38 −9 45)	0.77	0.67	−0.1	[−0.29; 0.09]	0.28	0.82
Superior frontal	R	(−20 27 23)	0.48	0.39	−0.09	[−0.26; 0.08]	0.29	0.82
Precentral gyrus	R	(−40 −10 45)	0.85	0.76	−0.09	[−0.27; 0.09]	0.30	0.82
Vermis	-	(−2 −59 −21)	0.51	0.42	−0.09	[−0.25; 0.08]	0.30	0.82
Supplementary motor area	L	(8 4 59)	0.66	0.57	−0.09	[−0.26; 0.08]	0.31	0.82
Cuneus	L	(8 −79 21)	1.04	0.94	−0.1	[−0.28; 0.1]	0.33	0.82
Putamen	R	(39 27 2)	1.27	1.16	−0.11	[−0.35; 0.13]	0.35	0.82
Inferior frontal	L	(−25 1 0)	0.59	0.5	−0.09	[−0.28; 0.1]	0.34	0.82
Cuneus	R	(−15 −78 24)	1.14	1.04	−0.09	[−0.32; 0.12]	0.35	0.82
Hippocampus/parahippocampal gyrus	L	(24 −19 −15)	0.01	0.09	0.08	[−0.1; 0.25]	0.39	0.86
Middle frontal	R	(−29 29 19)	0.88	0.79	−0.09	[−0.29; 0.12]	0.40	0.86
Precuneus	R	(−15 −59 37)	0.74	0.66	−0.08	[−0.25; 0.1]	0.41	0.86
Occipital lobe	R	(−34 −82 14)	0.88	0.8	−0.08	[−0.27; 0.12]	0.42	0.86
Gyrus rectus	R	(−11 38 −21)	0.71	0.64	−0.07	[−0.26; 0.12]	0.44	0.87
Supramarginal gyrus	R	(−49 −31 33)	0.77	0.7	−0.07	[−0.27; 0.13]	0.47	0.89
Temporal lobe	R	(−46 −25 −14)	0.51	0.45	−0.06	[−0.24; 0.11]	0.47	0.89
Heschl gyrus	R	(−36 −34 −26)	1.29	1.21	−0.08	[−0.35; 0.18]	0.51	0.90
Fusiform gyrus	R	(−51 −14 8)	0.48	0.42	−0.06	[−0.24; 0.12]	0.51	0.90
Calcarine fissure	R	(−17 −74 4)	1.23	1.16	−0.07	[−0.28; 0.15]	0.55	0.90
Inferior frontal	R	(−41 26 4)	0.64	0.59	−0.05	[−0.24; 0.14]	0.58	0.90
Pallidum	L	(17 −3 −2)	0.83	0.76	−0.07	[−0.33; 0.19]	0.59	0.90
Insula	L	(36 0 −1)	0.66	0.61	−0.05	[−0.24; 0.14]	0.59	0.90
Cerebellum crus	L	(28 −64 −36)	0.76	0.71	−0.05	[−0.24; 0.14]	0.60	0.90
Insula	R	(−38 1 −1)	0.73	0.69	−0.04	[−0.24; 0.14]	0.62	0.90
Cerebellum	R	(−55 −33)	0.83	0.88	0.05	[−0.16; 0.25]	0.64	0.90
Superior frontal	L	(17 30 25)	0.38	0.35	−0.03	[−0.2; 0.13]	0.66	0.90
Precuneus	L	(12 −59 39)	0.76	0.72	−0.04	[−0.23; 0.15]	0.69	0.90
Thalamus	L	(11 −20 6)	1.18	1.22	0.04	[−0.2; 0.29]	0.71	0.90
Calcarine fissure	L	(10 −75 2)	1.21	1.16	−0.05	[−0.27; 0.18]	0.71	0.90
Fusiform gyrus	L	(31 −40 −27)	0.48	0.44	−0.04	[−0.23; 0.16]	0.71	0.90
Thalamus	R	(17 −67 −7)	1.36	1.4	0.04	[−0.23; 0.33]	0.73	0.90
Lingual gyrus	L	(−53 −10 8)	0.96	0.92	−0.04	[−0.26; 0.18]	0.73	0.90
Rolandic operculum	R	(−13 −20 6)	0.5	0.47	−0.03	[−0.23; 0.17]	0.73	0.90
Paracentral lobule	R	(7 −12 41)	0.56	0.53	−0.03	[−0.21; 0.15]	0.74	0.90
Median cingulate cortex	L	(−8 −31 62)	0.87	0.83	−0.04	[−0.28; 0.2]	0.74	0.90
Occipital lobe	L	(30 −81 13)	0.78	0.75	−0.03	[−0.21; 0.15]	0.75	0.90
Angular gyrus	L	(43 −63 35)	0.83	0.8	−0.03	[−0.23; 0.17]	0.76	0.90
Temporal lobe	L	(44 −24 −13)	0.42	0.4	−0.02	[−0.21; 0.16]	0.78	0.92
Median cingulate cortex	R	(−9 −5 39)	1.08	1.05	−0.03	[−0.28; 0.22]	0.80	0.92
Gyrus rectus	L	(9 39 −21)	0.65	0.63	−0.02	[−0.22; 0.18]	0.82	0.93
Cerebellum	L	(25 −54 −32)	0.77	0.75	−0.02	[−0.2; 0.16]	0.84	0.94
Heschl gyrus	L	(48 −20 6)	1.27	1.29	0.02	[−0.27; 0.32]	0.87	0.94
Pallidum	R	(−21 −2 −2)	0.42	0.43	0.01	[−0.15; 0.17]	0.88	0.94
Hippocampus/parahippocampal gyrus	R	(−27 −13 −15)	0.04	0.05	0.01	[−0.16; 0.18]	0.89	0.94
Rolandic operculum	L	(49 −14 9)	0.52	0.51	−0.01	[−0.23; 0.21]	0.95	0.99
Amygdala	R	(−27 −1 −23)	0.04	0.03	−0.01	[−0.18; 0.17]	0.98	0.99
Cerebellum crus	R	(−32 −63 −37)	0.72	0.72	0	[−0.2; 0.2]	0.99	0.99
Amygdala	L	(21 −2 −22)	0.04	0.04	0	[−0.18; 0.17]	0.99	0.99

PET activity was normalized using the GM + WM activity for each subject. ROIs, regions of interest; TS, Tourette syndrome; R, right; L, left; two-sample t-tests: *Uncorrected *P*-value < 0.05; FDR-corrected *P*-value., false discovery rate *P*-value (corrected for multiple comparisons); CI, confidence interval.

### Associations between [^18^F]-FNM activity and clinical severity

**Figure 3:**
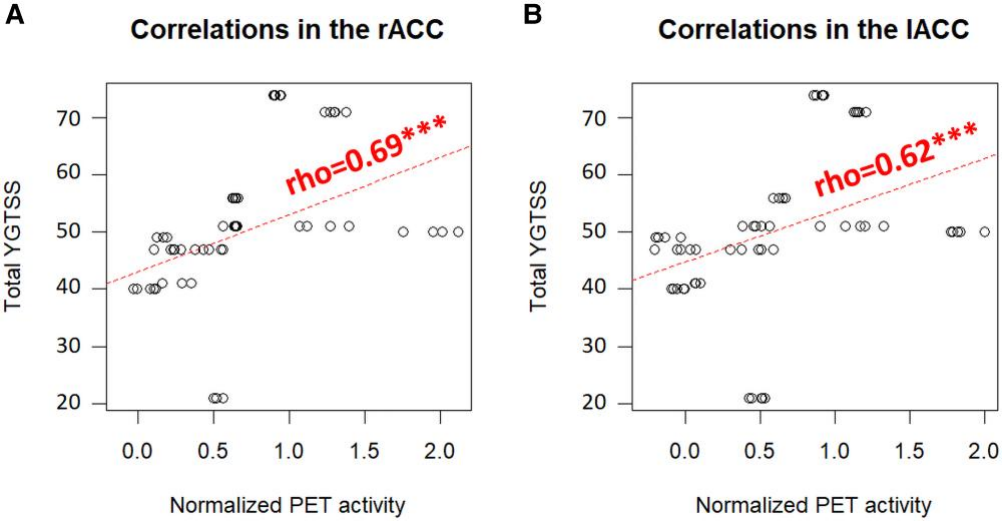
**Correlations between PET activity and clinical variables in patients with TS.** Correlations between the YGTSS scores and normalized PET activity in rACC **(A)** and the left anterior cingulate cortex (lACC) **(B)**. Each datapoint corresponds to one of the five values of the normalized PET activity of each patient with TS (*n* = 12 patients). Linear regressions in dotted lines. Rho , coefficient of correlations of Spearman; Spearman correlations: ***FDR-corrected *P*-value < 0.001.

We used these five ROIs (the rACC, the lACC, the right caudate nucleus, the right olfactory cortex and the left paracentral lobule) for the correlations between clinical/behavioural variables and normalized PET activity in patients with TS (a FDR correction was used to confirm significance). Only three correlations were both significant (FDR-corrected *P*-value < 0.05) and relevant (rho > |0.60|). Age was significantly and negatively correlated with the rACC activity (Spearman correlation: FDR *P*-value = 3 × 10^−6^ and rho=−63). The rACC and lACC activities were significantly and positively correlated with YGTSS total scores (Spearman correlations: FDR *P*-value = 6 × 10^−8^ and rho = 0.69; and FDR *P*-value = 3 × 10^−6^ and rho = 0.62, respectively). There were no significant and relevant correlations between the activity of the right caudate nucleus, nor the right olfactory cortex, nor the left paracentral lobule and clinical/behavioural variables, and no correlations with treatment dose. All correlations are presented in Supplementary Table 1, while the significant ones are presented in Table 3 and the main ones in Fig. 3.

**Table 3:** Significant correlations between normalized PET activity within specific ROIs and clinical/behavioural variables in patients with TS

ROI	Variable	Cor	*P*-value	FDR-corrected *P*-value
Right anterior cingulate cortex	Age (years)	**−0**.**63**	2E−07*	3E−06*
	YGTSS Total	**0**.**69**	2E−09*	6E−08*
	YGTSS Motor	0.58	2E−06*	3E−05*
	YGTSS Motor + Vocal	0.48	**0**.0002*	**0**.0008*
	YGTSS Impairment	0.57	3E−06*	3E−05*
	Y-BOCS Total	−0.30	**0**.02*	0.05
	Y-BOCS Obsessions	−0.41	**0**.001*	**0**.005*
	BIS-11 Motor impulsivity	0.54	1E−05*	9E−05*
	BIS-11 Cognitive impulsivity	0.38	**0**.004*	**0**.01*
Right caudate nucleus	Age (years)	−0.36	**0**.006*	**0**.02*
	Treatment dose (aripiprazole)	−0.28	**0**.03*	0.07
	YGTSS Total	0.51	5E−05*	**0**.0003*
	YGTSS Motor	0.47	**0**.0002*	**0**.0009*
	YGTSS Motor + Vocal	0.31	**0**.02*	**0**.04*
	YGTSS Impairment	0.42	**0**.0009*	**0**.004*
	Y-BOCS Obsessions	−0.31	**0**.02*	**0**.04*
	BIS-11 Motor impulsivity	0.34	**0**.009*	**0**.03*
Right olfactory cortex	Age (years)	−0.48	**0**.0001*	**0**.0006*
	Treatment dose (aripiprazole)	−0.30	**0**.02*	0.05
	YGTSS Total	0.57	3E−06*	3E−05*
	YGTSS Motor	0.49	1E−04*	**0**.0005*
	YGTSS Motor + Vocal	0.31	**0**.02*	5E−02*
	YGTSS Impairment	0.49	8E−05*	**0**.0005*
	Y-BOCS Total	−0.27	**0**.04*	0.08
	Y-BOCS Obsessions	−0.27	**0**.04*	0.09
	BIS-11 Motor impulsivity	0.38	**0**.003*	**0**.01*
Left anterior cingulate cortex	Age (in years)	−0.59	9E−07*	1E−05*
	YGTSS Total	**0**.**62**	2E−07*	3E−06*
	YGTSS Motor	0.57	**0**.000*	3E−05*
	YGTSS Motor + Vocal	0.49	**0**.00*	**0**.0005*
	YGTSS Impairment	0.47	**0**.0002*	**0**.0009*
	Y-BOCS Total	−0.33	**0**.01*	**0**.03*
	Y-BOCS Obsessions	−0.38	**0**.003*	**0**.01*
	BIS-11 Motor impulsivity	0.51	4E−05*	**0**.0003*
	BIS-11 Cognitive impulsivity	0.29	**0**.03*	0.06
Left paracentral lobule	Age (years)	−0.27	**0**.04*	0.08
	YGTSS Total	0.38	**0**.003*	**0**.01*
	YGTSS Motor	0.29	**0**.03*	0.06
	YGTSS Impairment	0.44	**0**.0006*	**0**.002*

ROI, region of interest; YGTSS, Yale Global Tic Severity Scale; Y-BOCS, Yale-Brown Obses*s*ive Compulsive Scale; HAD, Hospital Anxiety and Depression Scale; BIS-11, Barratt Impulsivity Scale-11 items; only significant correlations are presented here: coefficients of correlations (Cor) ≥ |0.6|and significant *P*-values (*P < 0.017) in bold; FDR-corrected *P*-value, false discovery rate *P*-value (corrected for multiple comparisons); significant FDR-corrected *P*-value (*FDR-P < 0.05) are also in bold.

## Discussion

In this discussion, we consider [^18^F]-FNM uptake as a marker of NMDAR activity in vivo due to its affinity and selectivity for open NMDAR.^38^ Greater radioligand binding represents larger NMDAR activations, which can be considered a consequence of higher glutamate release in the brain rather than simply an up-regulation of NMDARs as a compensatory phenomenon since uptake only reflects activated NMDARs.

This pilot imaging study evaluates the activity of NMADRs in vivo in patients with TS, using a new radiotracer (the [^18^F]-FNM). Patients with TS exhibited higher overall NMDAR activity than healthy controls and certain specific cerebral areas had even more activated NMDARs such as the bilateral ACC, the right caudate nucleus, the right olfactory cortex and the left paracentral lobule of patients with TS. Moreover, NMDAR activity in the right and left ACC was significantly linked with tics severity in patients with TS.

**Figure 4:**
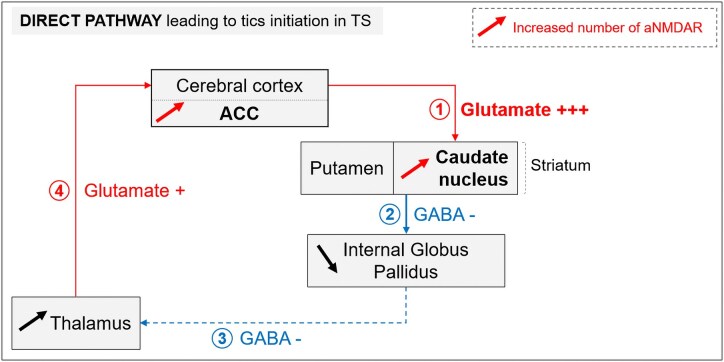
**Representation of the hypothesized disinhibition of the direct pathway in TS.** (1) Striatum activation by the cortex: the higher number of activated NMDARs observed in the caudate nucleus (part of the striatum) probably leads to an augmentation of its activity, (2) resulting in an accentuated release of GABA which inhibits the internal globus pallidus, (3) which in turn liberates less GABA causing a loss of inhibition of the thalamus, (4) which can therefore strongly activate the cerebral cortex initiating movements, where a higher number of activated NMDARs was observed in the ACC. This higher activity of the NMDARs in the caudate nucleus and ACC in patients with TS may result in tics emergence. aNMDAR, activated NMDARs, representing the presence of glutamate to activate NDMAR.

The ACC and the caudate nucleus are both parts of the CBGTC loops involved in TS,^8,45,46^ and a recent study, using magnetic resonance spectroscopy methodology, has similarly illustrated substantially increased striatal glutamate in TS children in the premotor cortex compared to controls.^47^ The caudate nucleus is implicated in both direct (initiating movements) and indirect (restricting movements) striatal pathways. It receives excitatory glutamatergic neurotransmission from the cortex, before inhibiting the internal part of the globus pallidus through a liberation of GABA in the direct pathway. Our results suggest that the caudate nucleus would be hyper-activated in patients with TS because of its hyper-NMDAR activity compared to controls. Hence, its hyper-activation would result in a hyper-inhibition of the internal part of the globus pallidus from the direct striatal pathway, which would lift the inhibition of the thalamus, resulting in higher excitatory afferent connections to the cortex and the ACC, probably occasioning the emergence of tics in TS (Fig. 4). Indeed, part of the limbic system, the ACC, is a central hub for the integration of several networks and plays a role in motor execution as well as vocalization, emotional processing etc.^48^ Forming part of the CBGTC loops described by Alexander *et al*. (1986), the ACC sends afferent connections to the ventral striatum and receives efferent ones from the thalamus.^49^ Therefore, the observed hyper-NMDAR activity within the ACC in patients with TS could be the consequence of the hyper-activated thalamus from the direct striatal pathway resulting in movement initiation such as motor tics. Our results are consistent with the literature suggesting that tics arise both as a result of increased activation of the direct striatal pathway due to hyper-glutamatergic activity and decreased activation of the indirect striatal pathway related to hypo-GABAergic activity.^32,50-53^ This model was validated using a NMDAR antagonist in the striatum, which reduced tics in mice and a GABA-A receptor agonist, which brought about the complete cessation of movements similar to tics.^22^ All these results combined tend to highlight the change in the balance between excitatory and inhibitory influences within the CBGTC loop in TS, with a reduced GABAergic inhibition and an increased glutamatergic activation in striatal pathways. Therefore, the observed increase in NMDAR activity in the caudate nucleus could be the consequence of a reduced GABAergic inhibition of glutamatergic pathways.

According to the literature, other hypotheses could be associated with the hyper-NMDAR activity observed here in the ACC of patients with TS. This may be related to impaired top-down control of motor pathways in TS as the ACC is involved in error monitoring and a recent review has suggested that hyperactivity of the dorsal ACC in OCD patients may impair goal-directed behaviour leading to obsessive-compulsive symptoms.^54^ A similar mechanism could be involved in the onset of tics, although in this study, behavioural data are insufficient to confirm this alteration in descending control. Nonetheless, two functional imaging studies have found increased activity within the ACC during motor and vocal tics in patients with TS.^45,55,56^ Consequently, TS symptoms could result of autonomous discharges from the ACC, with a lack of inhibition by the prefrontal cortices.^57^

An alternative theory would be that the supposed hyper-glutamatergic activity of the ACC in patients with TS would be linked to the inhibition of tics, rather than with their emergence, since patients were asked to remain still and to restrain their movements for the imaging procedure. For this scenario, several studies have shown an increased activity of the ACC during tics suppression in TS,^58,59^ indicating that fronto-striatal activation may help to maintain regulatory control over semi-involuntary behaviours.

In TS, abnormal connectivity and morphometric changes were observed in the paracentral lobule, as in other motor areas, and may be related to tics severity.^9,60-62^ Because of its connections with the primary motor cortex,^63^ the implication of the paracentral lobule within the motor loop is evident, and its hyper-NMDAR activity could result on its hyperactivation promoting tics’ emergence.

In regards to the olfactory cortex, sensory abnormalities were already reported in patients with TS such as the premonitory urge phenomena, enhanced sensory perception and altered olfaction,^64,65^ and brain activity was also impaired in the olfactory cortex in TS patients.^66^ Hence, we might suppose that a hyper-NMDAR activity in the olfactory cortex could be related to these sensory abnormalities in TS.

In terms of limitations, the observed difference between groups did not reach the FDR-corrected significance threshold. Nonetheless, it was a pilot study on a small number of subjects (two groups of *n* = 12) which aimed to find hints which would need to be replicated in a larger population. Moreover, our study only included adult patients with TS (≥18 years old) while TS is a neurodevelopmental disorder evolving during childhood around the age of 7 years^67,68^ and usually decreasing with age.^69,70^ Therefore, the observed hyper-NMDAR activity could be a result of this persistent TS: it could be an adaptive or mal-adaptive phenomenon specific to the chronification of TS, which may not be present in children with TS. Premonitory urges may also be related to increased activated NMDARs in patients with TS, but we were unable to verify this in this study because the absorption kinetics of the radioligand did not allow for it. Finally, we cannot rule out the effect of medication (all patients with TS were treated with aripiprazole), even if our analyses were adjusted with dose of treatment appropriately. A study reported that never-medicated patients with TS had lower concentrations of glutamine and glutamine–glutamate complex in the striatum versus controls while these concentrations increased after 4 weeks of aripiprazole treatment to reach similar levels between patients and controls.^71^ Similarly, decreased connectivity between the right caudate and the right insula in medicated patients with TS compared to unmedicated patients has been reported.^72^

Finally, we note that, in our study, patients with TS had few comorbidities (only 25% suffered from OCD (*n* = 3) and none suffered from ADHD). Thus, the observed NMDAR hyperactivity would be mainly related to tics and should be less affected by the presence of other forms of disinhibited behaviour.

In conclusion, this work has demonstrated implication of the glutamatergic system in the basal ganglia and the ACC that may underlie the emergence of tics. Nevertheless, several points will need to be clarified in the future, such as the higher NMDAR activity in TS children, the specific effect of anti-psychotic drugs on this glutamatergic dysfunction and the potential beneficial effect of anti-glutamatergic drugs.

## Supplementary Material

fcag212_Supplementary_Data

## Data Availability

The code has been supplied in the Supplementary material, as well as the files corresponding to the AAL atlas used in PMod (a nifti file to visualize the atlas and a text file presenting the ROIs). The rest of the data supporting the findings of this study are available on request from the corresponding author.
