# Supplementary material for: Tics severity in Tourette syndrome associated with higher glutamatergic activity in the anterior cingulate cortex
Source: Brain Commun. 2026 Jun 17;8(3):fcag212. doi: 10.1093/braincomms/fcag212 (PMC13281919; doi:10.1093/braincomms/fcag212)
Supplement: fcag212_Supplementary_Data [file fcag212_supplementary_data.zip › Supplementary data.docx]

**SUPPLEMENTARY DATA**

| **ROIs** | **Variables** | **Cor** | **p.value** |  | **FDR-corrected p.value** | |
| --- | --- | --- | --- | --- | --- | --- |
| **Right Anterior Cingulate Cortex (rACC)** | **Age** *(in years)* | **-0.63** | **2E-07** | * | **3E-06** | ***** |
|  | **Treatment dose (aripiprazole)** | -0.26 | 0.05 |  | 0.10 |  |
|  | **YGTSS Total** | **0.69** | **2E-09** | * | **6E-08** | ***** |
|  | **YGTSS Motor** | 0.58 | **2E-06** | * | **3E-05** | ***** |
|  | **YGTSS Vocal** | 0.16 | 0.23 |  | 0.35 |  |
|  | **YGTSS Motor + Vocal** | 0.48 | **0.0002** | * | **0.0008** | ***** |
|  | **YGTSS Impairment** | 0.57 | **3E-06** | * | **3E-05** | ***** |
|  | **Y-BOCS Total** | -0.30 | **0.02** | * | 0.05 |  |
|  | **Y-BOCS Obsessions** | -0.41 | **0.001** | * | **0.005** | ***** |
|  | **Y-BOCS Compulsions** | -0.12 | 0.37 |  | 0.50 |  |
|  | **HAD Total** | 0.06 | 0.65 |  | 0.76 |  |
|  | **HAD Anxiety** | 0.06 | 0.66 |  | 0.77 |  |
|  | **HAD Depression** | 0.13 | 0.35 |  | 0.47 |  |
|  | **BIS-11 Total** | 0.23 | 0.08 |  | 0.15 |  |
|  | **BIS-11 Planning difficulties** | -0.02 | 0.87 |  | 0.92 |  |
|  | **BIS-11 Motor impulsivity** | 0.54 | **1E-05** | * | **9E-05** | ***** |
|  | **BIS-11 Cognitive impulsivity** | 0.38 | **0.004** | * | **0.01** | ***** |
| **Right Caudate Nucleus** | **Age** *(in years)* | -0.36 | **0.006** | * | **0.02** | ***** |
|  | **Treatment dose (aripiprazole)** | -0.28 | **0.03** | * | 0.07 |  |
|  | **YGTSS Total** | 0.51 | **5E-05** | * | **0.0003** | ***** |
|  | **YGTSS Motor** | 0.47 | **0.0002** | * | **0.0009** | ***** |
|  | **YGTSS Vocal** | 0.07 | 0.58 |  | 0.71 |  |
|  | **YGTSS Motor + Vocal** | 0.31 | **0.02** | * | **0.04** | ***** |
|  | **YGTSS Impairment** | 0.42 | **0.0009** | * | **0.004** | ***** |
|  | **Y-BOCS Total** | -0.23 | 0.09 |  | 0.16 |  |
|  | **Y-BOCS Obsessions** | -0.31 | **0.02** | * | **0.04** | ***** |
|  | **Y-BOCS Compulsions** | -0.10 | 0.44 |  | 0.57 |  |
|  | **HAD Total** | -0.01 | 0.92 |  | 0.97 |  |
|  | **HAD Anxiety** | 0.10 | 0.45 |  | 0.58 |  |
|  | **HAD Depression** | -0.14 | 0.31 |  | 0.44 |  |
|  | **BIS-11 Total** | 0.04 | 0.77 |  | 0.85 |  |
|  | **BIS-11 Planning difficulties** | -0.22 | 0.10 |  | 0.19 |  |
|  | **BIS-11 Motor impulsivity** | 0.34 | **0.009** | * | **0.03** | ***** |
|  | **BIS-11 Cognitive impulsivity** | 0.21 | 0.11 |  | 0.20 |  |
| **Right Olfactory cortex** | **Age** *(in years)* | -0.48 | **0.0001** | * | **0.0006** | ***** |
|  | **Treatment dose (aripiprazole)** | -0.30 | **0.02** | * | 0.05 |  |
|  | **YGTSS Total** | 0.57 | **3E-06** | * | **3E-05** | ***** |
|  | **YGTSS Motor** | 0.49 | **1E-04** | * | **0.0005** | ***** |
|  | **YGTSS Vocal** | 0.05 | 7E-01 |  | 0.82 |  |
|  | **YGTSS Motor + Vocal** | 0.31 | **0.02** | * | **5E-02** | ***** |
|  | **YGTSS Impairment** | 0.49 | **8E-05** | * | **0.0005** | ***** |
|  | **Y-BOCS Total** | -0.27 | **0.04** | * | 0.08 |  |
|  | **Y-BOCS Obsessions** | -0.27 | **0.04** | * | 0.09 |  |
|  | **Y-BOCS Compulsions** | -0.16 | 0.22 |  | 0.35 |  |
|  | **HAD Total** | 0.03 | 0.85 |  | 0.92 |  |
|  | **HAD Anxiety** | 0.06 | 0.68 |  | 0.78 |  |
|  | **HAD Depression** | 0.06 | 0.64 |  | 0.76 |  |
|  | **BIS-11 Total** | 0.04 | 0.77 |  | 0.85 |  |
|  | **BIS-11 Planning difficulties** | -0.15 | 0.25 |  | 0.36 |  |
|  | **BIS-11 Motor impulsivity** | 0.38 | **0.003** | * | **0.01** | ***** |
|  | **BIS-11 Cognitive impulsivity** | 0.17 | 0.21 |  | 0.35 |  |
| **Left Anterior Cingulate Cortex (lACC)** | **Age** *(in years)* | -0.59 | **9E-07** | * | **1E-05** | ***** |
|  | **Treatment dose (aripiprazole)** | -0.16 | 0.23 |  | 0.35 |  |
|  | **YGTSS Total** | **0.62** | **2E-07** | * | **3E-06** | ***** |
|  | **YGTSS Motor** | 0.57 | **0.000** | * | **3E-05** | ***** |
|  | **YGTSS Vocal** | 0.19 | 0.15 |  | 0.25 |  |
|  | **YGTSS Motor + Vocal** | 0.49 | **0.00** | * | **0.0005** | ***** |
|  | **YGTSS Impairment** | 0.47 | **0.0002** | * | **0.0009** | ***** |
|  | **Y-BOCS Total** | -0.33 | **0.01** | * | **0.03** | ***** |
|  | **Y-BOCS Obsessions** | -0.38 | **0.003** | * | **0.01** | ***** |
|  | **Y-BOCS Compulsions** | -0.16 | 0.23 |  | 0.35 |  |
|  | **HAD Total** | -0.03 | 0.83 |  | 0.90 |  |
|  | **HAD Anxiety** | 0.00 | 1.00 |  | 1.00 |  |
|  | **HAD Depression** | 0.01 | 0.94 |  | 0.98 |  |
|  | **BIS-11 Total** | 0.16 | 0.24 |  | 0.36 |  |
|  | **BIS-11 Planning difficulties** | -0.09 | 0.50 |  | 0.64 |  |
|  | **BIS-11 Motor impulsivity** | 0.51 | **4E-05** | * | **0.0003** | ***** |
|  | **BIS-11 Cognitive impulsivity** | 0.29 | **0.03** | * | 0.06 |  |
| **Left Paracentral lobule** | **Age** *(in years)* | -0.27 | **0.04** | * | 0.08 |  |
|  | **Treatment dose (aripiprazole)** | -0.20 | 0.14 |  | 0.24 |  |
|  | **YGTSS Total** | 0.38 | **0.003** | * | **0.01** | ***** |
|  | **YGTSS Motor** | 0.29 | **0.03** | * | 0.06 |  |
|  | **YGTSS Vocal** | -0.04 | 0.79 |  | 0.86 |  |
|  | **YGTSS Motor + Vocal** | 0.13 | 0.34 |  | 0.47 |  |
|  | **YGTSS Impairment** | 0.44 | **0.0006** | * | **0.002** | ***** |
|  | **Y-BOCS Total** | -0.18 | 0.17 |  | 0.29 |  |
|  | **Y-BOCS Obsessions** | -0.18 | 0.17 |  | 0.29 |  |
|  | **Y-BOCS Compulsions** | 0.00 | 0.99 |  | 1.00 |  |
|  | **HAD Total** | 0.08 | 0.56 |  | 0.69 |  |
|  | **HAD Anxiety** | 0.10 | 0.43 |  | 0.57 |  |
|  | **HAD Depression** | 0.09 | 0.52 |  | 0.65 |  |
|  | **BIS-11 Total** | -0.07 | 0.59 |  | 0.71 |  |
|  | **BIS-11 Planning difficulties** | -0.18 | 0.18 |  | 0.29 |  |
|  | **BIS-11 Motor impulsivity** | 0.16 | 0.23 |  | 0.35 |  |
|  | **BIS-11 Cognitive impulsivity** | 0.01 | 0.97 |  | 0.99 |  |

**Supplementary Table 1. Correlations between normalised PET activity within specific ROIs and clinical/behavioural variables in patients** **with TS.**

*ROIs = Regions of Interest; YGTSS = Yale Global Tic Severity Scale; Y-BOCS = Yale-Brown Obsessive Compulsive Scale; HAD = Hospital Anxiety and Depression Scale; BIS-11 = Barratt Impulsivity Scale-11 items; Coefficients of Correlations (Cor) ≥ |0.6|and significant p-values (p < 0.017) in bold; FDR-corrected p.value = False Discovery Rate p-value (corrected for multiple comparisons); significant (FDR-corrected p.value < 0.05) and relevant (Cor ≥ |0.6|) correlations are in bold and highlighted in grey.*

*
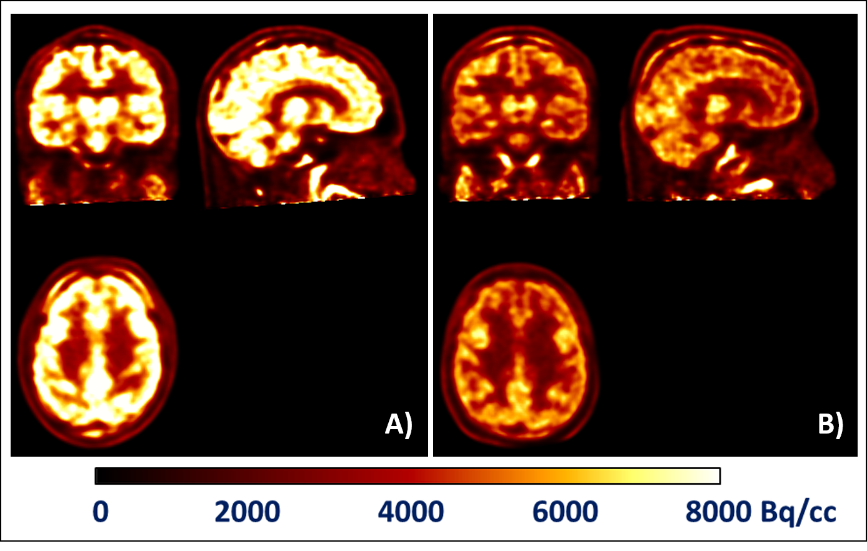
*

**Supplementary Figure 1. Comparisons of PET activity between two paired subjects.**

*PET cerebral imaging of* ***A)*** *a patient* *with TS and* ***B)*** *its paired healthy control in a normalised template. Scale from dark red to light represents the intensity of PET uptake, which is the intensity of NMDAR activity in PET imaging for both subjects in Bq/cc. The injected weight-to-dose ratio was of 3.4 MBq/kg for the patient with TS and 3.6 MBq/kg for its paired healthy control. Frames A and B: x, y, z = 81, 113, 153. ANOVA of the difference between groups (n=12 patients with TS versus n=12 healthy controls): p=1.2 x 10^-5^, F=19.12 and df between groups=1.*
